# Advances in diabetic foot ulcer management: a narrative review of novel antibiotics, nanotechnology, and regenerative therapeutics

**DOI:** 10.3389/fmicb.2026.1882663

**Published:** 2026-10-13

**Authors:** Mohd Saleem, Farida Habib Khan, Sumayyah Mohammad Almarshedy, Fahaad S. Alenazi, Hamad Alshetaiwi, Irfan Ahmad, Fauwaz Fahad Alrashid, Syed Muhammad. Ozair Ilyas, Abdulrahman Moussa Almuwaisa, Wael Nahi Alnomasi, Mohd Shahid Khan

**Affiliations:** 1 Department of Pathology, College of Medicine, University of Hail, Hail, Saudi Arabia; 2 Department of Family and Community Medicine, College of Medicine, University of Hail, Hail, Saudi Arabia; 3 Department of Medicine, College of Medicine, University of Hail, Hail, Saudi Arabia; 4 Department of Pharmacology, Medical Education Committee, College of Medicine, University of Hail, Hail, Saudi Arabia; 5 Department of Clinical Laboratory Sciences, College of Applied Medical Sciences, King Khalid University, Abha, Saudi Arabia; 6 Health and Medical Research Center, King Khalid University, Abha, Saudi Arabia; 7 Department of Surgery, College of Medicine, University of Hail, Hail, Saudi Arabia; 8 Department of General Surgery, King Khalid Hospital, Hail, Saudi Arabia; 9 Department of Microbiology, Hind Institute of Medical Sciences, Sitapur, Uttar Pradesh, India

**Keywords:** biofilm, diabetic foot ulcer, multidrug-resistant infections, nanotechnology-based drug delivery, wound healing therapies

## Abstract

Diabetic foot ulcers (DFUs) are a major cause of hospitalization, chronic infection, and non-traumatic lower-limb amputation, imposing substantial clinical and economic burdens worldwide. Standard DFU management requires a multidisciplinary approach involving pressure offloading, regular debridement, infection control, metabolic optimisation, vascular assessment, and revascularisation when indicated. Offloading and sharp debridement reduce mechanical stress, remove devitalized tissue, disrupt biofilms, and support wound healing. Infected ulcers require appropriate microbiological assessment and culture-directed antimicrobial therapy, particularly when multidrug-resistant (MDR) pathogens are present. Biofilm formation is an important feature of chronic DFUs and is associated with persistent infection, delayed healing, and treatment failure. Biofilm-associated antimicrobial tolerance results from factors including reduced antimicrobial penetration, metabolic dormancy, altered growth states, and phenotypic heterogeneity, and should be distinguished from genetically mediated antimicrobial resistance caused by acquired or chromosomal genetic determinants. Although biofilm-associated tolerance does not necessarily involve genetic resistance, persistent biofilm infection may facilitate the selection and persistence of resistant subpopulations. Both Gram-positive and Gram-negative organisms can form biofilms in DFUs. Novel antibiotics, including oxazolidinones, lipoglycopeptides, lipopeptides, and advanced β-lactam/β-lactamase inhibitor combinations, may provide options for selected difficult to treat infections, although evidence varies among agents and clinical settings. Adjunctive wound-care strategies, including appropriate dressings and negative-pressure wound therapy, may support wound-bed preparation and healing in selected cases. Emerging approaches such as nanoparticle-based delivery systems, hydrogels, nanofibrous scaffolds, stem-cell and exosome therapies, gene and RNA-based strategies, and oxygen-releasing biomaterials have shown potential in preclinical and early clinical studies. However, their clinical effectiveness, safety, reproducibility, scalability, and regulatory approval and clinical translation remain incompletely established. Integrating evidence-based standard care with appropriate antimicrobial therapy and carefully evaluated emerging technologies may facilitate individualized management of complex DFUs.

## Introduction

Diabetes mellitus (DM) has emerged as one of the most significant and rapidly growing global health challenges. The International Diabetes Federation (IDF) Diabetes Atlas 2025 estimates that about 589 million adults aged 20–79 years currently live with diabetes, representing about 11.1% of the world’s adult population (Duncan et al., 2025). This number is projected to increase to approximately 853 million by 2050, reflecting the continuing global diabetes epidemic driven by population growth, urbanization, sedentary lifestyles, and increasing obesity. More than 80% of people with diabetes live in low and middle-income countries, where limited access to healthcare services and preventive care further increases the risk of diabetes-related complications (Duncan et al., 2025).

One of the most serious and frequent complications of diabetes is diabetic foot ulcer (DFU). A large meta-analysis estimated the global prevalence of DFU among individuals with diabetes at approximately 6.3%, with substantial geographic variation, while the lifetime risk of developing a DFU has been estimated at 19–34% (Zhang et al., 2017). Globally, approximately 18.6 million people are estimated to develop a DFU annually. DFUs are associated with substantial morbidity and are implicated in approximately 80% of non-traumatic lower-extremity amputations among patients with diabetes (Armstrong et al., 2023).

The clinical consequences of DFUs extend beyond ulcer development. Infection is reported in approximately 50–60% of DFUs and contributes substantially to hospitalization, amputation, and mortality. In patients with moderate-to-severe DFUs, a considerable proportion may ultimately require lower-extremity amputation, while mortality is substantially higher among patients with DFUs than among those with diabetes without foot complications (Armstrong et al., 2023). Recurrence is also a major clinical challenge, with reported recurrence rates reaching approximately 65% within 5 years, highlighting the chronic and recurrent nature of DFUs (De Souza Santos et al., 2025).

The economic burden of DFUs is similarly substantial, encompassing costs associated with hospitalization, wound care, antimicrobial therapy, surgical procedures, rehabilitation, and lower-extremity amputation. The burden is particularly pronounced in settings where preventive services, early detection, multidisciplinary foot-care programs, and specialized wound-care facilities are limited (Alaydrus et al., 2025). Thus, the global burden of DFUs provides an important context for understanding the challenges faced by regions with a high prevalence of diabetes and diabetes-related complications.

The Middle East represents one such region, with a disproportionately high burden of diabetes and its associated complications. According to the IDF, approximately 73 million adults in the Middle East and North Africa (MENA) region are living with diabetes, placing the region among those with the highest diabetes burden globally (Sun et al., 2022). Within the Gulf Cooperation Council (GCC) countries, diabetes prevalence has been reported to be substantially higher than in non-GCC Middle Eastern countries and is projected to increase further in the coming decades (Alessa et al., 2025). The growing prevalence of diabetes, together with prolonged disease duration and associated metabolic and vascular complications, is likely to increase the regional burden of diabetic foot disease (DFD).

DFUs are also an important and insufficiently characterized complication of diabetes in Middle Eastern countries. A systematic review of Arab countries reported DFU prevalence estimates ranging from 2.7% in Iraq to 11.85% in Saudi Arabia, with estimates of 4.2% in Egypt, 4.65% in Jordan, and 5.9% in Bahrain (Mairghani et al., 2017). These findings indicate substantial heterogeneity in the regional burden, although the available evidence remains limited and unevenly distributed across countries. Furthermore, a study involving patients with type 2 diabetes in Qatar, Kuwait, and Saudi Arabia reported that 2.9% had an active DFU, while more than half were considered at risk of developing DFU because of neuropathy and other associated complications (Ponirakis et al., 2022).

The development and management of DFUs in the Middle East are influenced by a combination of demographic, metabolic, socioeconomic, cultural, and healthcare-related factors. Rapid urbanization, increasing obesity, sedentary lifestyles, and dietary changes have contributed to the growing prevalence of diabetes and its complications in the region (Aljulifi, 2021; Meo et al., 2019). In addition, limited awareness of foot-care practices, delayed healthcare-seeking behavior, traditional treatment practices, socioeconomic constraints, poor living conditions, and inadequate access to multidisciplinary diabetic foot services may contribute to delayed presentation and poorer outcomes (Alessa et al., 2025; Mairghani et al., 2017). These region-specific factors highlight the need for management strategies that are not only clinically effective but also appropriate for the healthcare, socioeconomic, and cultural context of Middle Eastern populations.

Despite the increasing burden of DFUs, substantial gaps remain in their prevention and management, particularly in settings with limited healthcare resources. In addition, socioeconomic, cultural, and healthcare-related factors may influence the development, management, and outcomes of DFUs and warrant greater consideration in clinical practice. Therefore, this narrative review aims to summarize the current evidence on advances in DFU management, with particular emphasis on novel antibiotics, nanotechnology-based approaches, regenerative therapeutics, and their potential clinical applications. The review also highlights current challenges and research gaps, with particular consideration of issues relevant to the Middle Eastern region, to identify strategies that may improve clinical outcomes and reduce DFU-related morbidity, amputation, and healthcare burden.

## Literature search

A literature search was conducted in PubMed/MEDLINE, Scopus, and Web of Science for studies published from 2010 to 2026. Major search terms included “diabetic foot ulcer,” “diabetic foot infection,” “wound healing,” “antibiotics,” “antimicrobial resistance,” “biofilm,” “novel antibiotics,” “nanotechnology,” “nanoparticles,” “hydrogels,” “regenerative medicine,” “stem cells,” “exosomes,” and “growth factors,” combined using Boolean operators (AND/OR). For the regional component, terms related to the Middle East, Gulf Cooperation Council, and individual Middle Eastern countries were also included.

Relevant original studies, clinical trials, systematic reviews, meta-analyses, narrative reviews, guidelines, and consensus documents were considered based on their relevance to DFU epidemiology, microbiology, antimicrobial management, advanced wound care, nanotechnology, and regenerative therapies. Duplicate and irrelevant publications were excluded, and reference lists of key articles were screened to identify additional relevant studies. As this was a narrative review, study selection was based on relevance to the review objectives rather than a formal systematic-review screening process.

### Pathophysiology and predisposing factors of diabetic foot ulcers

DFUs arise from a complex interplay of systemic and local factors (Table 1).

**Table 1:** Predisposing factors for diabetic foot ulceration.

Systemic factor	Local factor	Ref.
Uncontrolled hyperglycaemia	Diabetic peripheral neuropathy	Khan et al. (2017)
Long diabetes duration (>10 years)	Foot deformities	Khan et al. (2017)
Peripheral arterial disease	Trauma / ill-fitted footwear	Khan et al. (2017)
Retinopathy	Prior ulcer or amputation	Khan et al. (2017)
Chronic nephropathy	Callus formation	Khan et al. (2017)
Advanced age	High plantar pressure	Khan et al. (2017)
Elevated BMI	Limited mobility	Khan et al. (2017)

DFUs are caused mainly by diabetic neuropathy, peripheral arterial disease (PAD), as well as immune failure. Neuropathy (up to 70% of patients) is a dysfunction that affects nociception and proprioception, which causes the development of repetitive trauma, structural malformations, and the development of ulcers (Doğruel et al., 2022). The assessment of neuropathy usually involves the evaluation of the small and large fiber function through pinprick, thermal sensation, vibration sensation, and the 10 g monofilament test (American Diabetes Association, 2021). PAD is common in patients with DFUs and substantially increases the risk of ischemia and impaired wound healing. Clinical assessment includes evaluation of symptoms such as intermittent claudication, rest pain, and reduced walking capacity, together with vascular examination, including palpation of the dorsalis pedis and posterior tibial pulses, assessment of skin temperature, color, and trophic changes. The ankle-brachial index (ABI) is commonly used for non-invasive assessment of PAD; an ABI of approximately 0.9–1.3 is generally considered within the normal range, whereas values <0.9 suggest PAD and values >1.3 may indicate poorly compressible arteries due to medial arterial calcification (Hinchliffe et al., 2020; Kurze et al., 2022). In patients with diabetes, ABI should therefore be interpreted cautiously. Additional vascular assessment may include toe-brachial index (TBI), toe pressure, and Doppler ultrasonography. Doppler arterial waveforms are typically described as triphasic, biphasic, or monophasic, with loss of normal triphasic flow suggesting arterial disease (Hinchliffe et al., 2020; Kurze et al., 2022). Microvascular dysfunction may persist despite apparently preserved macrovascular perfusion and can be assessed using techniques such as laser Doppler flowmetry. Hyperglycaemia also compromises the immunity of the innate and adaptive types, making it more prone to infection via several mechanisms such as T-lymphocyte apoptosis, imbalance of pro-inflammatory cytokines, neutrophil impairment, and disruption of keratinocyte migration (Reardon et al., 2020; Aumiller and Dollahite, 2015). The prolonged hyperglycaemia (≥150 mg/dL) facilitates bacterial growth, especially aerobic Gram-positive microorganisms, and impairs the activity of fibroblasts, collagen production, and tissue regeneration (Misbah Mehraj, 2018).

### Infection in diabetic foot ulcers

DFI is a major complication of DFUs and is associated with substantial morbidity and an increased risk of lower-extremity amputation. Although DFUs are frequently colonized by microorganisms, microbial colonization should not be considered synonymous with clinical infection. Colonization refers to the presence of microorganisms in the ulcer without associated tissue invasion or a host inflammatory response, and therefore bacterial load alone does not establish DFI. In contrast, DFI is a clinical diagnosis, characterized by evidence of local or systemic inflammation resulting from microbial invasion of tissues. According to the Infectious Diseases Society of America (IDSA)/International Working Group on the Diabetic Foot (IWGDF) criteria, infection is diagnosed clinically when at least two local manifestations, such as erythema, induration, warmth, pain or tenderness, or purulent discharge, are present (Pereira et al., 2017). Important factors predisposing to DFI and impairing wound healing include ischemia, peripheral neuropathy, oedema, and impaired host defense. The wound microbiome may influence disease progression and chronicity; however, the mere detection or quantification of microorganisms does not distinguish colonization from infection. PAD and associated endothelial dysfunction, including impaired nitric oxide-mediated vasodilation, may further compromise microcirculation, aggravate tissue ischemia, and increase the risk of ulcer progression and infection (Baig et al., 2022).

### Microbial distribution in DFUs: global and Middle East

DFUs may harbor a diverse range of microorganisms, but the reported microbial spectrum varies considerably according to geography, ulcer severity and chronicity, prior antimicrobial exposure, and the method used for specimen collection and microbiological identification. Importantly, microorganisms recovered from an ulcer do not necessarily represent the organisms responsible for clinical infection. Therefore, interpretation of microbial-distribution studies requires consideration of the distinction between colonization and clinically diagnosed DFI.

*Staphylococcus aureus* is consistently reported among the most frequently isolated pathogens from infected DFUs worldwide. However, Gram-negative bacilli, including *Pseudomonas aeruginosa*, *Escherichia coli*, and other Enterobacterales, are also frequently reported, particularly in chronic, deep, previously treated, or complicated infections and in several low and middle-income settings, including parts of the Middle East, Asia, and Africa (Garousi et al., 2023). The relative contribution of these organisms differs substantially between studies and geographic regions. A systematic review reported *P. aeruginosa* in approximately 16.6–18.5% of DFU infections overall, although markedly higher proportions have been reported in individual cohorts, particularly in some Asian settings (Garousi et al., 2023). Such variability may reflect differences in climate and environmental exposure, healthcare practices, patient characteristics, ulcer severity, antimicrobial exposure, and microbiological methods.

The method of specimen collection is an important determinant of the organisms recovered. Superficial wound swabs are easy to obtain but may recover colonizing microorganisms and may not accurately represent pathogens present in deeper infected tissues. Current IWGDF/IDSA guidelines therefore recommend, when culture is indicated, obtaining an aseptic tissue specimen by curettage or biopsy after appropriate wound cleansing and debridement. Studies summarized in these guidelines indicate that tissue specimens have greater sensitivity and specificity than superficial swabs for identifying causative microorganisms (Senneville et al., 2024). Differences in specimen collection, processing, transport, culture conditions, and organism-identification methods may further contribute to the heterogeneity of reported microbial profiles (Senneville et al., 2024; Lipsky et al., 2012).

Ulcer severity and clinical characteristics also influence the microbial profile. Superficial and less complicated infections may be dominated by Gram-positive cocci, particularly *S. aureus* and streptococci, whereas chronic, deep, necrotic, ischemic, or previously treated ulcers are more likely to yield Gram-negative bacilli and, in selected cases, anaerobic organisms. Polymicrobial infection is particularly common in chronic or deep DFIs, although the reported frequency varies according to the definition of infection and the sampling strategy used. Prior antibiotic exposure can additionally suppress susceptible organisms and select for resistant or less readily cultured pathogens, thereby altering the spectrum recovered by culture.

Methicillin-resistant *Staphylococcus aureus* (MRSA) is also reported in DFIs worldwide, but its prevalence varies substantially by region and study population. Meta-analytic data have estimated a global prevalence of approximately 17% among DFU isolates, with higher proportions reported in some regions than others (Zhou et al., 2024). Regional differences in antimicrobial use, healthcare-associated transmission, infection-control practices, and local resistance patterns may contribute to this heterogeneity.

Similar variation has been reported in the Middle East. A culture-based study from Kuwait found both Gram-positive and Gram-negative organisms among infected DFUs, with Gram-positive organisms more frequently associated with non-ischemic ulcers and Gram-negative organisms more frequently recovered from ischemic or complicated wounds (Alhubail et al., 2020). However, such findings should be interpreted in the context of the sampling approach and patient population, as differences in specimen quality and prior antimicrobial exposure may influence the organisms recovered.

Overall, the microbial spectrum of clinically infected DFUs includes both Gram-positive and Gram-negative organisms, with *S. aureus*, *P. aeruginosa*, *E. coli*, and other Enterobacterales frequently reported. Nevertheless, there is no single universal microbial profile for DFIs. Geographic setting, ulcer characteristics and severity, prior antibiotic exposure, and especially the method of specimen collection can substantially influence reported pathogen distributions. Consequently, microbiological findings are best interpreted using appropriately collected specimens, preferably deep tissue obtained after wound preparation and debridement in clinically infected ulcers, rather than relying solely on superficial swab cultures. This methodological and regional heterogeneity supports the use of local microbiological data to inform culture-directed and region-specific antimicrobial management of DFIs.

### Multidrug-resistant and biofilm phenotypes of diabetic foot ulcers

Formation of biofilms is a key pathogenic process in DFUs and is closely associated with chronicity, delayed healing, and treatment failure. Microorganisms within the wound microenvironment adhere to surfaces and become embedded within an extracellular matrix composed of polysaccharides, proteins, and extracellular DNA, forming structured communities that promote persistence and evasion of host defenses (Aishwarya et al., 2025). Biofilm formation can increase antimicrobial tolerance and bacterial persistence through reduced antimicrobial penetration, metabolic dormancy, altered growth states, and phenotypic heterogeneity. These mechanisms should be distinguished from genetically mediated antimicrobial resistance, which results from stable genetic determinants such as resistance genes or mutations. Although biofilm-associated tolerance may allow bacteria to survive antimicrobial exposure without acquiring genetic resistance, prolonged biofilm-associated infection may facilitate the selection and persistence of genetically resistant subpopulations.

Both Gram-positive and Gram-negative organisms can form biofilms in DFUs, with species-specific variation. *Staphylococcus aureus* has been reported as one of the most frequent biofilm-forming organisms (approximately 38.8%), followed by *Pseudomonas aeruginosa*, *Citrobacter* spp., *Escherichia coli*, and *Proteus* spp. (approximately 10.5% each) (Banu et al., 2015). Biofilm formation may act in concert with neuropathy, repetitive trauma, structural deformities, and disruption of the tissue barrier to promote persistent infection and tissue damage (Baig et al., 2022; Figure 1).

**Figure 1:**
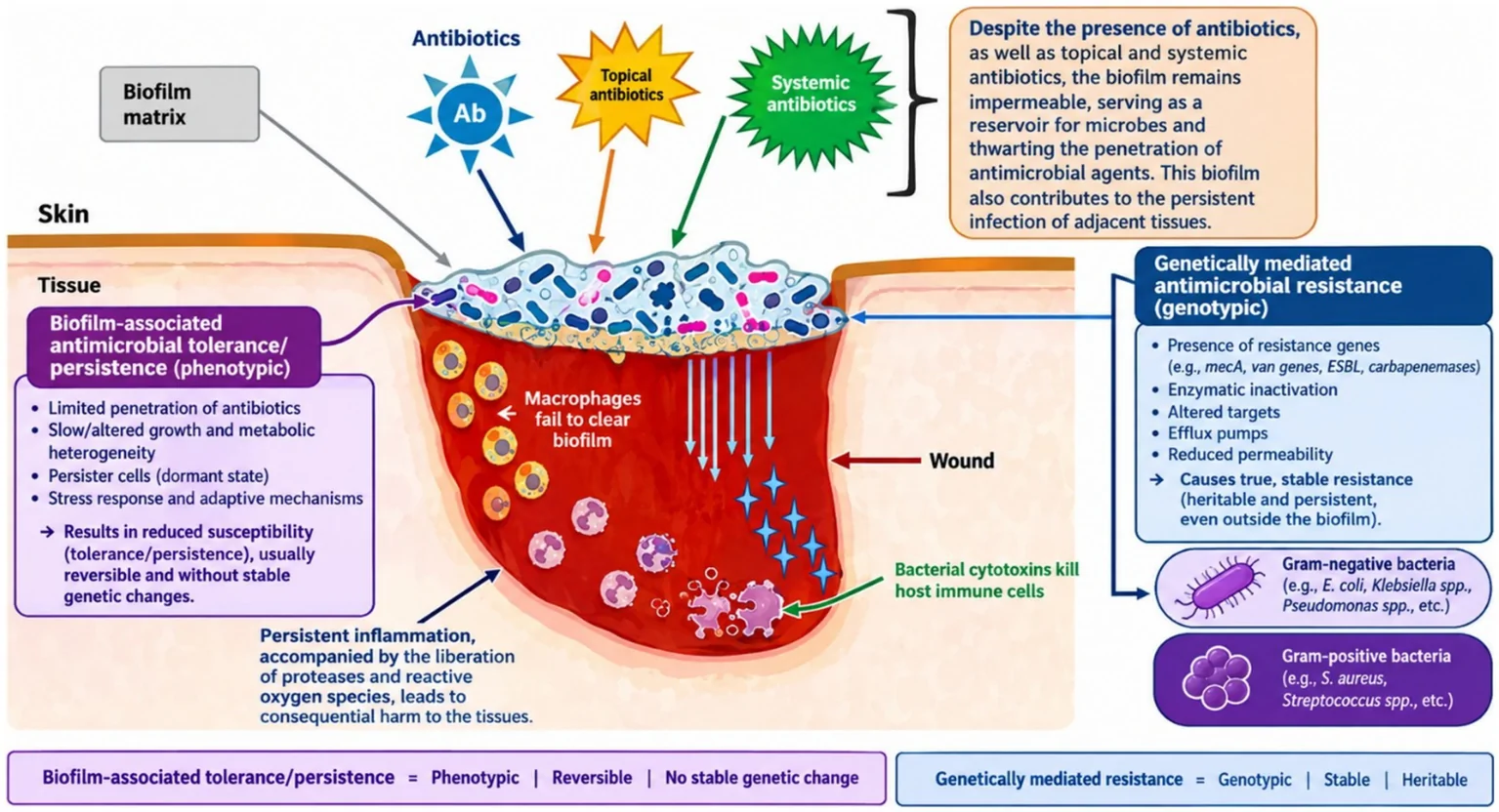
Mechanisms of Biofilm-Mediated Recalcitrance, Host Immune Evasion, and Tissue Damage in Chronic Wounds. (The extracellular polymeric substance (EPS) biofilm matrix acts as a physical barrier, restricting the penetration of both topical and systemic antibiotics while shielding encapsulated polymicrobial communities (including Gram-positive pathogens such as *S. aureus* and *Streptococcus* spp., as well as Gram-negative pathogens such as *Pseudomonas* spp., *E. coli*, and *Klebsiella* spp.). Encapsulated bacteria exhibit phenotypic antimicrobial tolerance and persistence, driven by matrix diffusion limits, metabolic heterogeneity, nutrient gradients, and the presence of non-dividing persister cells; this tolerance is reversible and non-heritable upon dispersion. Concurrently, bacteria may harbor genetically mediated antimicrobial resistance (e.g., mecA, van genes, ESBLs, carbapenemases) conferring stable, heritable resistance via enzymatic cleavage, target modification, efflux pump upregulation, or reduced membrane permeability. The persistent biofilm construct frustrates host immune responses, preventing effective phagocytosis by macrophages. Secreted bacterial cytotoxins directly kill infiltrating immune cells, fuelling a cycle of unresolving inflammation characterized by excessive release of host proteases and reactive oxygen species (ROS) that exacerbate adjacent tissue destruction).

The increasing prevalence of multidrug-resistant organisms (MDROs) complicates the management of DFIs and is associated with inappropriate antimicrobial use, including unnecessary, excessive, or inadequate treatment. Antibiotics should be reserved for clinically infected ulcers, with empirical therapy guided by infection severity and local resistance patterns, followed by culture-directed treatment and antimicrobial susceptibility testing according to Clinical and Laboratory Standards Institute (CLSI) or European Committee on Antimicrobial Susceptibility Testing (EUCAST) standards. Antibiotic therapy is not indicated for uninfected ulcers or for routine prophylaxis, as unnecessary exposure may promote antimicrobial resistance (Jouhar et al., 2020).

In Saudi Arabia and the wider Middle East, high rates of antimicrobial resistance have been reported among DFIs. In one Saudi cohort, 54% of infections were polymicrobial, and 61.5% of isolates produced biofilm; previous amputation, polymicrobial infection, recent antibiotic exposure, and longer ulcer duration were associated with resistant infection (Saleem et al., 2024). Among the *Staphylococcus aureus* isolates in this study, MRSA resistance was associated with the presence of *mecA*, which was detected in 81.5% of isolates, providing a genetic basis for methicillin resistance (Saleem et al., 2024). Among Gram-negative isolates, ESBL production was predominantly associated with *blaCTX-M*, while carbapenem resistance was reported in 25.6% of isolates and was mainly associated with *blaNDM* (Saleem et al., 2024). These findings illustrate the importance of linking resistance genes to both the organism and the corresponding resistance phenotype.

In another DFU study involving 200 bacterial isolates, 51.7% were carbapenemase producers, with *blaKPC* and *blaNDM* reported as the predominant carbapenemase genes (Saleem et al., 2025). Carbapenemase-producing organisms were associated with severe infection and polymicrobial growth and were reported in patients with higher rates of minor and major amputation, emphasizing their clinical importance (Saleem et al., 2025).

Internationally, MDR, polymicrobial infection, and biofilm formation are also frequently reported in DFIs. Prospective cohorts have reported polymicrobial infection rates of approximately 33–43%, whereas biofilm production has been reported in approximately 53–71% of isolates and has frequently been associated with MDR phenotypes (Khan et al., 2025a; Saleem et al., 2025). In larger series, overall MDR prevalence has approached 67%, with Gram-negative organisms such as *Escherichia coli*, *Pseudomonas aeruginosa*, and *Proteus mirabilis* frequently contributing to the MDR burden. Among Gram-positive organisms, resistant *S. aureus*, including MRSA (51.8%), and vancomycin-resistant enterococci (33.3%) have also been reported (Khan et al., 2025a; Saleem et al., 2025). ESBL production has been observed in approximately 31.1% of Gram-negative isolates, with *blaCTX-M* representing an important mechanism of resistance (Khan et al., 2025a; Saleem et al., 2025).

Several clinical factors, including prolonged ulcer duration, neuropathy, tobacco exposure, previous antimicrobial therapy, polymicrobial infection, and biofilm formation, have been associated with MDR infection and adverse clinical outcomes (Khan et al., 2025b). MDR and carbapenem-resistant infections may contribute to prolonged hospitalization, treatment failure, and increased risk of amputation. These findings highlight the need for antimicrobial stewardship, appropriate microbiological sampling, routine susceptibility testing, and local surveillance to guide empirical and definitive antimicrobial therapy (Jouhar et al., 2020; Khan et al., 2025b).

### Therapeutic modalities for diabetic foot ulcer

#### Standard of care (foundation)

DFUs management is based on an evidence-based, multidisciplinary methodology and aims at addressing issues that slow down healing of wounds and preventing complications (infection and amputation). The main components of the standard of care are pressure off-loading, wound debridement, infection control, metabolic optimization, and vascular assessment and revascularization (Table 2).

**Table 2:** Core components of standard of care in DFU management.

Component	Key Interventions	Rationale/Clinical benefit	Ref.
Pressure off-loading	Total contact cast (TCC); non-removable knee-high walkers; removable cast walkers when contraindicated; therapeutic footwear for prevention	Redistributes plantar pressure, reduces repetitive trauma, accelerates ulcer healing, and lowers recurrence rates	Randel (2016); Khan et al. (2024)
Debridement	Regular sharp/surgical debridement; removal of necrotic tissue and callus; adjunct enzymatic/autolytic methods when required	Converts chronic wound to an acute healing state; reduces bacterial burden and biofilm; promotes granulation and epithelialization	Khan et al. (2024)
Infection control	Deep tissue culture; severity-based systemic antibiotics; drainage of abscess; surgical debridement for moderate–severe infection	Prevents progression to osteomyelitis/sepsis; reduces amputation risk; ensures targeted antimicrobial therapy	Randel (2016)
Metabolic optimization	Tight glycemic control; management of hypertension, dyslipidemia, nutrition; smoking cessation; patient education	Improves immune function, tissue perfusion, and wound healing; reduces recurrence and complications	Randel (2016); Khan et al. (2024)
Vascular assessment and revascularization	ABI/TBI/Doppler evaluation; endovascular angioplasty/stenting; surgical bypass when indicated	Restores tissue perfusion; essential for healing ischemic ulcers; improves limb salvage and decreases major amputation	Khan et al. (2024)
Multidisciplinary care	Coordinated management by diabetology, surgery, vascular, infectious disease, podiatry, wound care teams	Associated with faster healing, fewer hospitalizations, and reduced amputation rates	Khan et al. (2024)

### Adjunctive local and systemic therapeutic strategies in DFU management

#### Wound dressings

Wound dressings form a vital part of the DFU treatment, as they play a protective and a curative role. They serve a shield against environmental pollutants, thus preventing secondary infection, as well as keeping the physiological environment moist, which encourages autolytic debridement, endogenous enzyme activity and granulation tissue development and re-epithelialization (Bakker et al., 2012). The dressing selection must be customized based on the volume of exudates on the wound and tissue nature. Heavy exuding ulcers also necessitate absorbent dressing, including alginate or foam dressing to maintain excess moisture and avoid skin maceration of the surrounding skin. On the other hand, dry or slightly exudative wounds are favourable to moisture containing hydrogel or hydrocolloids that help in maintaining hydration and healing. The proper choice of dressing, consequently, has a direct impact on wound development and clinical outcome (Everett and Mathioudakis, 2018).

#### Negative pressure wound therapy

NPWT may be considered as an adjunctive treatment for selected diabetes-related foot wounds, particularly complex or post-operative wounds, when standard wound care alone is insufficient. NPWT applies controlled sub-atmospheric pressure to the wound bed and may facilitate exudate management, reduce oedema, promote granulation tissue formation, and support wound contraction. However, its benefits depend on appropriate patient selection, wound preparation, pressure settings, and professional application. Current evidence provides greater support for NPWT in post-operative diabetic foot wounds, whereas evidence for its routine use in non-surgical chronic DFUs remains less certain (Everett and Mathioudakis, 2018; Zaver and Kankanalu, 2023). NPWT should therefore be used as part of comprehensive wound management, including debridement, infection control, offloading, and assessment of perfusion, rather than as a replacement for these measures. Treatment duration should be individualized according to wound characteristics and clinical response.

#### Topical therapies

Topical antimicrobial and antiseptic preparations are not routinely recommended to promote healing of diabetes-related foot ulcers. Current IWGDF guidance recommends against the routine use of topical antiseptic or antimicrobial dressings for wound healing because available evidence has not demonstrated consistent benefit over standard care and the certainty of evidence is limited (Lipsky et al., 2020). Their use should therefore not substitute for appropriate debridement, wound cleansing, offloading, infection management, or systemic antimicrobial therapy when clinically indicated. Selected topical antiseptics may have a role in specific clinical circumstances, but their use should be individualized and guided by the wound condition and local practice. Emerging topical therapies, including novel agents investigated in clinical trials, remain investigational or adjunctive, and evidence should be interpreted cautiously. For example, although a phase III randomized controlled trial of topical esmol reported improved healing compared with standard wound care, further independent evidence is required before routine use can be recommended (Rastogi et al., 2023).

#### Systemic antibiotic therapy

Systemic antibiotics should be used only for clinically diagnosed DFI and should be selected according to the severity of infection, likely pathogens, previous antimicrobial exposure, local resistance patterns, microbiological findings, and patient-specific factors. Uninfected DFUs should not be treated with systemic antibiotics (Rastogi et al., 2023).

For mild DFI, treatment is generally appropriate in the outpatient setting with oral agents targeting the likely Gram-positive pathogens, particularly *Staphylococcus aureus* and *streptococci*. Depending on local resistance patterns and patient factors, commonly used options include cephalexin, dicloxacillin, amoxicillin-clavulanate, or clindamycin. When MRSA is suspected or confirmed, agents such as trimethoprim-sulfamethoxazole, doxycycline/minocycline, clindamycin, or linezolid may be considered according to susceptibility results (Rastogi et al., 2023; Khan et al., 2024).

Moderate and severe DFIs require broader empirical coverage when indicated, with the spectrum determined by infection severity, previous microbiological results, prior antibiotic exposure, and local epidemiology. Severe infections generally require hospitalization and intravenous therapy, with empirical regimens selected to cover Gram-positive and, when clinically indicated, Gram-negative and anaerobic pathogens. Broader-spectrum β-lactams, β-lactam/β-lactamase inhibitor combinations, cephalosporins, or carbapenems may be used in selected patients, with additional MRSA coverage when risk factors are present (Khan et al., 2024).

The duration of antibiotic therapy should be individualized according to the severity and clinical response of the infection, extent of tissue involvement, and presence or absence of osteomyelitis. According to the 2023 IWGDF/IDSA recommendations, antibiotic therapy for diabetic foot soft-tissue infection should generally be administered for 1–2 weeks. Treatment may be extended to up to 3–4 weeks when the infection is extensive, is resolving more slowly than expected, or when severe PAD is present. Following surgical debridement of moderate or severe soft-tissue infection, a shorter course may be appropriate in selected patients. For diabetic foot osteomyelitis, antibiotic therapy may be given for up to 3 weeks after minor amputation when the bone-margin culture is positive, whereas 6 weeks is recommended for osteomyelitis treated without bone resection or amputation (Senneville et al., 2024; Bronze Michael Stuart Diabetic Foot Infections Medication, 2020). Antibiotic therapy should be reviewed when microbiological results become available and de-escalated to the narrowest effective agent whenever appropriate. Culture-directed treatment and antimicrobial stewardship are essential to minimize unnecessary broad-spectrum exposure, adverse effects, and selection of antimicrobial resistance (Khan et al., 2024; Bronze Michael Stuart Diabetic Foot Infections Medication, 2020).

#### Surgical management

DFU care may need surgical intervention when conservative measures fail or when there is a complication. The procedures can be categorized as either vascular or non-vascular (Khan et al., 2024). Vascular interventions such as angioplasty, stenting or bypass grafting are meant to restore normal perfusion in patients with peripheral arterial disease and are important to facilitate wound healing. Non-vascular surgery is performed to repair structural defects, to excise infected or necrotic tissue and to resolve biomechanical defects; this can be reconstructive, prophylactic, curative or emergent, based on clinical conditions (Khan et al., 2024). Amputation is a final therapeutic intervention which occurs in severe tissue necrosis, uncontrolled infections or life-threatening complications, especially when the ulcer is in Wagner grade 4 or 5. However, it can be concluded that early surgical decision-making can decrease morbidity and increase the results of limb salvage (Musuuza et al., 2020).

### Role of novel antibiotics in the management of DFIs

DFIs are clinically challenging because of their variable severity, polymicrobial etiology in many moderate and severe infections, chronic wound environment, and increasing prevalence of antimicrobial resistance. Although several newer antimicrobial agents provide activity against MDR pathogens or offer pharmacokinetic advantages, their role in DFIs should be interpreted according to the strength and type of available clinical evidence (Alenazi and Khan, 2025). The 2023 IWGDF/IDSA guideline recommends selecting antimicrobial therapy based on likely or proven pathogens and their susceptibility, infection severity, published evidence of efficacy in DFIs, adverse-effect profile, drug interactions, antimicrobial stewardship considerations, availability, and cost. Importantly, no antibiotic class or individual agent has shown consistent overall superiority for treating DFIs (Senneville et al., 2024).

Among agents with established clinical experience in DFIs, linezolid and daptomycin remain important options for infections caused by resistant Gram-positive organisms, particularly MRSA. Linezolid inhibits bacterial protein synthesis by binding to the 50S ribosomal subunit and has demonstrated efficacy in diabetic foot and complicated skin and soft-tissue infections. However, prolonged treatment requires monitoring for adverse effects, including hematological toxicity and peripheral or optic neuropathy. Daptomycin causes rapid, calcium-dependent disruption of the bacterial cytoplasmic membrane and is active against MRSA and vancomycin-resistant enterococci (VRE). It may be useful for severe Gram-positive DFI and diabetic foot osteomyelitis, although creatine phosphokinase monitoring is required (Hashemian et al., 2018; Beiras-Fernandez et al., 2010).

Ceftaroline represents a newer β-lactam with specific activity against MRSA through high-affinity binding to penicillin-binding protein 2a. Evidence directly relevant to DFIs includes observational data from the CAPTURE registry, in which ceftaroline was associated with clinical success in patients with DFIs, including infections involving MRSA and mixed pathogens (Lipsky et al., 2015). Nevertheless, these data are predominantly observational rather than derived from contemporary randomized trials specifically designed to establish the superiority of ceftaroline in DFIs. Thus, ceftaroline can be considered an option for selected Gram-positive DFIs when its spectrum and susceptibility profile are appropriate, rather than as a universally preferred treatment.

Long-acting lipoglycopeptides, particularly dalbavancin and oritavancin, provide prolonged antimicrobial exposure and infrequent dosing and may therefore have practical advantages in selected patients requiring prolonged parenteral therapy. However, their evidence base in DFIs remains substantially smaller than that for established agents. Dalbavancin has been evaluated mainly in diabetic foot osteomyelitis and other deep infections through observational studies and limited clinical investigations, with reported favourable outcomes in selected patients (Núñez et al., 2025). These findings suggest a potential role in carefully selected Gram-positive infections, particularly when prolonged outpatient intravenous therapy is difficult, but they should not be interpreted as evidence of superior efficacy in routine DFI management. Evidence for oritavancin in DFIs is even more limited, and its use should therefore be considered largely extrapolative.

Tedizolid, another oxazolidinone, provides once-daily administration and has a potentially more favourable toxicity profile than linezolid during relatively prolonged treatment. However, its clinical evidence in DFIs is limited compared with linezolid. Consequently, although its pharmacological properties may be advantageous in selected circumstances, the available evidence does not support considering tedizolid an established first-line treatment for DFIs (Moran et al., 2014; Bouza et al., 2018).

Newer agents directed against resistant Gram-negative organisms, including ceftazidime-avibactam, ceftolozane-tazobactam, cefiderocol, meropenem-vaborbactam, and imipenem-cilastatin-relebactam, have expanded therapeutic options for infections caused by selected ESBL-producing organisms and carbapenem-resistant Gram-negative bacteria. Their principal clinical evidence, however, derives from complicated urinary tract infections, hospital-acquired or ventilator-associated pneumonia, bloodstream infections, and other serious infections rather than from adequately powered DFI-specific randomized controlled trials. Consequently, their use in DFIs should generally be reserved for culture-confirmed or strongly suspected infections caused by susceptible resistant Gram-negative organisms for which conventional agents are unsuitable. Their inclusion in DFI treatment should therefore be regarded as targeted or extrapolated therapy rather than evidence-based routine treatment (Senneville et al., 2024; Infectious Diseases Society of America, 2026).

Cefiderocol is of particular interest because its siderophore-mediated mechanism facilitates bacterial iron-transport-dependent entry and provides activity against several highly resistant Gram-negative pathogens. Nevertheless, clinical experience specifically in DFIs remains limited, and available evidence has largely been generated in other serious infections. Similarly, ceftazidime-avibactam and ceftolozane-tazobactam may be valuable for selected resistant Gram-negative infections, but their use should be guided by microbiological results and local resistance patterns rather than by the assumption that newer agents are inherently superior to conventional therapy (Mougakou et al., 2023; Kaye et al., 2023).

Overall, novel antibiotics should be viewed as an important component of the expanding antimicrobial collection rather than as replacements for established DFI therapies. Their principal value lies in providing additional options when resistant pathogens, antimicrobial intolerance, drug interactions, or difficulties with prolonged intravenous therapy limit conventional treatment. The strength of evidence varies substantially among agents: linezolid, daptomycin, and ceftaroline have relevant clinical experience in DFI or closely related skin and soft-tissue infections; dalbavancin has emerging evidence particularly in diabetic foot osteomyelitis; whereas several newer anti-Gram-negative agents remain supported predominantly by evidence from other serious infections. Accordingly, treatment should remain individualized and integrated with appropriate source control, wound care, vascular assessment, microbiological diagnosis, and antimicrobial stewardship.

### Recent advances in the field of DFU treatment

Nano-drug delivery systems (NDDS) are emerging approaches that use nanoscale materials to improve the stability, localization, and controlled release of therapeutic agents. In DFUs, where impaired angiogenesis, persistent inflammation, oxidative stress, and altered extracellular matrix remodeling contribute to delayed healing, NDDS have been investigated as platforms for the localized delivery of bioactive molecules and other therapeutic agents (Liu et al., 2023). These systems may facilitate controlled and sustained release and potentially improve the therapeutic availability of agents that are limited by instability, short half-life, or poor local retention.

However, the evidence supporting many NDDS-based interventions in DFU management remains predominantly preclinical or at an early translational stage. Most reported studies have evaluated these approaches using *in vitro* experiments or diabetic animal models, and their observed effects should therefore not be interpreted as established clinical efficacy.

### Bioactive molecules

Growth factors and other bioactive molecules play important roles in cellular proliferation, differentiation, angiogenesis, and tissue remodeling during wound healing. Key mediators include TGF-β, VEGF, PDGF, EGF, and FGF2. In chronic diabetic wounds, dysregulation of these pathways may contribute to impaired tissue repair, creating a rationale for localized and controlled delivery of selected growth factors (Borena et al., 2015; Apte et al., 2019; Johnson and Wilgus, 2014; Haase et al., 2003). NDDS-based delivery platforms have therefore been investigated to improve the spatial and temporal availability of growth factors and enhance their biological effects. The studies summarized in Table 3 represent preclinical evidence, comprising *in vitro* and animal studies; no human clinical evidence was identified among these growth-factor nanocarrier studies.

**Table 3:** Preclinical studies of nanocarrier-based growth-factor delivery in diabetic wound healing.

Growth factors (GFs)	GF-Carriers	Function	Advantage	Evidence level	Ref.
EGF	Chitosan nanoparticle	Stimulate complete reformation of the epithelial layer, ensure an ample deposition of collagen, and expedite the maturation of collagen.	Favourable biocompatibility	Preclinical (*in vitro* + animal)	Lee et al. (2021)
EGF	PHBV-GelMA hybrid patch	Facilitates the movement and growth of diverse cell types, including keratinocytes, fibroblasts, and endothelial cells, while also promoting angiogenesis.	Favourable biostability	Preclinical (in vitro + animal)	Augustine et al. (2021)
Bfgf	Acellular dermal matrix	Strengthen the formation of granulation tissue, promote angiogenesis, and facilitate the deposition of collagen.	Effective stimulation of endothelial cells.	Preclinical (in vitro + animal)	Shi et al. (2021)
EGF	Chitosan/PVA hetero-composite hydrogel	Diminish the inflammatory response, expedite collagen deposition, and promote enhanced collagen maturation.	Introduce EGF and PHMB within a milieu abundant in ions.	Preclinical (in vitro + animal)	Lee and Lin (2022)
PDGF-BB	Nanohydrogel	Eradicate the biofilm.	Disrupt the biofilm while maintaining its structural integrity at room temperature.	Preclinical (in vitro + animal)	Santhini et al. (2022)

GF, growth factor; EGF, epidermal growth factor; bFGF, basic fibroblast growth factor; PDGF-BB, platelet-derived growth factor-two B subunits; PHBV-GelmA, Poly (3-hydroxybutyrate-co-3-hydroxyvalerate)- Gelatin-methacryloyl; PVA, polyvinyl alcohol gel; PHMB, polyhexamethylene biguanide.

EGF exhibits strong mitogenic activity on epithelial, fibroblastoid, and endothelial cells. However, the DFU microenvironment is characterized by impaired growth-factor signaling, including reduced EGF production and receptor expression. The short half-life of EGF and the need for repeated administration are additional limitations of conventional EGF therapy (Tuyet et al., 2009). Early clinical evidence suggests potential benefit of topical recombinant human EGF in DFUs. In a preliminary pilot study involving 28 patients, topical recombinant human EGF (0.005% Easyef) was associated with progressive wound closure, with complete healing observed in 13 of 23 patients who completed the 8-week follow-up (Tuyet et al., 2009). However, the small sample size and preliminary study design limit the strength of these findings, and larger controlled clinical studies are required to establish its efficacy and safety.

Biomaterial-based approaches have subsequently been investigated to improve growth-factor retention and sustained delivery in diabetic wounds. For example, Shi et al. developed a book-shaped acellular dermal matrix incorporating adipose-derived stromal cell sheets and sustained-release basic fibroblast growth factor (bFGF), which promoted diabetic wound healing in a preclinical model (Shi et al., 2021). Unlike the EGF study, this approach represents preclinical evidence and should not be presented as human clinical evidence. Overall, growth-factor-based therapies show translational potential, but the available evidence varies substantially by growth factor and delivery platform.

### Gene/protein or peptides

Gene and nucleic-acid-based approaches have emerged as potential strategies for modifying impaired molecular pathways involved in diabetic wound healing. MicroRNAs (miRNAs) regulate post-transcriptional gene expression, and miR-129 and miR-335 have been shown to target specificity protein-1 (Sp1), thereby inhibiting Sp1-mediated MMP-9 expression and promoting diabetic wound healing in experimental models (Wang et al., 2018). However, the clinical translation of gene-based therapies remains limited by challenges related to delivery efficiency, stability, off-target effects, and safety.

NDDSs have been investigated to improve the stability, cellular uptake, and delivery of therapeutic nucleic acids. Yan et al. used milk-derived exosomes to deliver miR-31-5p and demonstrated enhanced cellular uptake and resistance to degradation, together with improved endothelial-cell functions *in vitro* and enhanced angiogenesis and diabetic wound healing in mice (Yan et al., 2022). Similarly, Shaabani et al. developed a layer-by-layer siRNA delivery platform targeting prolyl hydroxylase domain 2 (PHD-2), thereby promoting HIF-1α stabilization and increasing proangiogenic factors in experimental diabetic wound conditions (Shaabani et al., 2021). These findings provide promising preclinical evidence for miRNA and siRNA-based approaches; however, their therapeutic efficacy and safety have not yet been established in adequately powered human clinical trials for DFUs.

Table 4 shows that other nanocarrier-based approaches have investigated the delivery of VEGF-A mRNA, CCN1, P311 peptides, bioactive peptides, and miR-146a using various nanoparticle-based systems. These studies have reported effects on angiogenesis, inflammation, oxidative stress, and wound healing. However, the available evidence is predominantly preclinical, with no established human clinical evidence for these specific platforms in DFUs. Further studies are required to confirm their safety and clinical efficacy.

**Table 4:** Preclinical nanocarrier-based delivery of therapeutic proteins, peptides, and nucleic acids for diabetic wound regeneration.

Therapeutic cargo	Carrier	Function	Evidence level	Ref.
VEGF-A mRNA	Ionizable lipid-mediated nanoparticles	Enhances VEGF-A expression and promotes wound healing	Preclinical (in vitro + animal)	Zha et al. (2023)
CCN1	Nanoparticle formulation	Increases intracellular CCN1 expression and reduces inflammation	Preclinical (in vitro + animal)	Jiang et al. (2022)
P311 peptide	ROS-responsive micelles	Enables ROS-triggered P311 release and promotes re-epithelialization under oxidative stress	Preclinical (in vitro + animal)	Shi et al. (2022)
Bioactive peptides	Chitosan nanoparticles	Modulates inflammation and promotes neovascularization and tissue regeneration	Preclinical (in vitro + animal)	Nanditha and Kumar (2022)
miR-146a	Cerium oxide nanoparticles	Modulates oxidative stress and inflammation and improves impaired wound healing	Preclinical (in vitro + animal)	Dewberry et al. (2022)
miR-31-5p	Milk-derived exosomes	Promotes endothelial-cell proliferation, migration, and angiogenesis	Preclinical (in vitro + animal)	Yan et al. (2022)

VEGF-A mRNA, vascular endothelial growth factor-A mRNA; miRNA, microRNA; NPs, nanoparticles; CCN 1, cellular communication network factor 1; NFκB, nuclear factor (κB).

### Stem cell/exosomes

Mesenchymal stem cells (MSCs), particularly adipose-derived stem cells (ADSCs), have attracted interest in regenerative medicine because of their regenerative and immunomodulatory properties (Costa et al., 2021). However, challenges including limited cell survival, storage and transportation requirements, and potential safety concerns may restrict their clinical translation. Biomaterial-assisted approaches, including injectable ADSC-containing hydrogels, have been investigated to improve cell retention and tissue repair under diabetic wound conditions (Jin et al., 2020).

Exosomes, which are small extracellular vesicles released by cells, have also been investigated as cell-free alternatives to stem-cell therapy. For example, Pluronic F127 hydrogel loaded with human umbilical cord MSC-derived exosomes (hUCMSC-exos) has demonstrated enhanced tissue regeneration and wound healing in diabetic wound models (Yang et al., 2020). These findings provide promising preclinical evidence; however, stem-cell, exosome, and hydrogel-based approaches remain experimental, with limited human clinical evidence in DFUs. Further clinical studies are required to establish their safety, optimal delivery, and therapeutic efficacy. This method is very promising in the treatment and repair of cutaneous wounds (regenerative medicine) (Table 5).

**Table 5:** Nanomaterial-based delivery systems for stem cells, conditioned media, and exosomes in diabetic wound regeneration.

Stem cells/Exosomes/Secretome	Animal model	Delivery system	Function	Evidence level	Ref.
hUCMSC-derived exosomes (hUCMSC-exos)	STZ-induced diabetic rats	Pluronic F-127 (PF-127) hydrogel	Accelerates wound closure and promotes granulation tissue regeneration and angiogenesis	Preclinical (in vitro + animal)	Yang et al. (2020)
Adipose-derived stem cells (ADSCs)	Diabetic rats	Injectable hypoxia-induced conductive hydrogel	Injectable hypoxia-induced conductive hydrogel	Preclinical (in vitro + animal)	Jin et al. (2020)
MSCs	Diabetic animal model	EGF-curcumin bandage bioconjugate (EGF-Cur B)	Promotes wound closure, granulation tissue formation, collagen deposition, and angiogenesis	Preclinical (in vitro + animal)	Mohanty and Pradhan (2020)
hFDSPC-conditioned medium (hFDSPC-CM)	db/db diabetic mice	Hyaluronic acid (HA) hydrogel	Promotes re-epithelialization, angiogenesis, collagen regeneration and maturation, and reduces inflammation	Preclinical (in vitro + animal)	Xin et al. (2021)
MSCs	STZ-induced diabetic mice	PLGA@IL-8 nanoparticles-loaded acellular dermal matrix	Enhances MSC survival, proliferation and endothelial differentiation and promotes capillary formation, collagen deposition, and wound healing	Preclinical (in vitro + animal)	Zhang et al. (2023)

hUCMS-exos, human umbilical cord-derived mesenchymal stem cell-derived exosomes; ADSCs, adipose-derived stem cells; BMSCs, bone marrow stem cells; hFDSPC, human foreskin-derived dermal stem/progenitor cell; MSCs, mesenchymal stem cells; STZ, streptozotocin; HA, hyaluronic acid; PLGA NPs, poly (lactic-co-glycolic acid)-nanoparticles.

### Drugs: nanoscale drug delivery solutions for wound healing

Several therapeutic agents have limited utility in chronic wound management because of poor aqueous solubility, chemical instability, limited tissue penetration, short residence time, or unfavorable pharmacokinetic properties. Nanoscale drug-delivery systems have therefore been investigated as a means of improving local retention, controlled release, stability, and bioavailability. However, these platforms should be regarded as investigational delivery strategies rather than comprehensive solutions, and most evidence in diabetic wound healing remains preclinical.

Curcumin, a natural polyphenol derived from *Curcuma longa*, has antioxidant and anti-inflammatory properties, but its poor solubility, stability, and bioavailability limit its therapeutic application. Nanoformulation can improve these physicochemical characteristics and facilitate localized and sustained delivery. Liu et al. developed curcumin nanoparticles encapsulated in gelatin microspheres and incorporated them into a thermosensitive hydrogel. The formulation was designed to provide wound-responsive release and enhanced local delivery of curcumin and promoted wound healing in an experimental diabetic wound model (Liu et al., 2018). Similarly, Hu et al. developed a hyaluronic acid/chitosan-based hydrogel for the delivery of curcumin nanoparticles, with controlled release and enhanced antioxidant and anti-inflammatory activity (Hu et al., 2021). These findings support the potential of nano-enabled curcumin delivery; however, the available evidence is predominantly experimental, and clinical efficacy and long-term safety remain to be established.

Antidiabetic agents, including metformin, pioglitazone, and glibenclamide, have also been investigated for their potential effects on inflammatory and regenerative pathways involved in diabetic wound healing. Cam et al. incorporated these agents into electrospun nanofibrous scaffolds based on different polymeric composites, including CS/GEL/PCL and PVP/PVL, and reported enhanced wound healing in experimental diabetic models (Cam et al., 2021). Other studies evaluating pioglitazone-containing fibrous formulations and combinations of pioglitazone with metformin or glibenclamide have reported improved wound-healing outcomes compared with pioglitazone alone (Cam et al., 2020). These studies provide preclinical evidence for localized drug delivery, but they should not be interpreted as evidence of established clinical efficacy in patients with DFUs.

### Non-bioactive substances

#### Metal-ion nanoparticles

Metal-based nanoparticles, including silver (Ag), gold (Au), and copper (Cu), have been investigated as antimicrobial components of advanced wound-dressing systems. However, their potential role should not be considered a substitute for conventional antimicrobial therapy in clinically infected diabetic foot ulcers. Their biological activity depends on factors such as particle size, morphology, surface characteristics, concentration, and ion release, and their safety requires careful evaluation.

Silver-based nanomaterials have demonstrated broad-spectrum antibacterial activity against clinically relevant and multidrug-resistant bacteria. For example, ampicillin–silver nanoformulations exhibited antibacterial activity against multidrug-resistant bacterial isolates (Khatoon et al., 2019). Wang et al. developed virus-like mesoporous silica-coated silver nanocubes with enhanced bacterial adhesion and incorporated the nanostructure into a hydrogel dressing; the formulation demonstrated antibacterial activity and improved wound healing in an experimental diabetic wound model (Wang et al., 2021). These findings provide preclinical support for the incorporation of silver-based nanomaterials into antimicrobial wound-dressing systems.

Gold nanoparticles have also demonstrated antimicrobial activity against multidrug-resistant bacteria (Li et al., 2014). However, the study cited here primarily provides antimicrobial evidence rather than direct evidence of diabetic wound-healing efficacy. Therefore, the potential application of gold nanoparticles in diabetic wound management should be considered investigational rather than clinically established. Copper nanoparticles have similarly been incorporated into hydrogels with photothermal antibacterial activity and wound-healing effects in experimental models (Tao et al., 2019). These findings indicate potential multifunctional applications of metal-based nanomaterials, but their clinical utility remains to be established.

Importantly, translation of metal-based nanomaterials into routine diabetic wound care requires evaluation of dose-dependent cytotoxicity, tissue accumulation, ion release, long-term biocompatibility, formulation stability, manufacturing reproducibility, scalability, sterilization, and regulatory requirements. Consequently, the available evidence should be interpreted as predominantly preclinical, and well-designed human studies are required to establish their safety and clinical effectiveness in diabetic foot ulcers.

#### Oxygen supply

Persistent tissue hypoxia is an important feature of chronic diabetic wounds and can adversely affect angiogenesis, cellular proliferation, and tissue repair (Hong et al., 2014). Conventional advanced dressings, including Hydrofiber®-based products, primarily regulate wound moisture and exudate and should not be considered oxygen-delivery therapies (Huang et al., 2021). More recently, nanotechnology-based oxygen-generating systems have been investigated to provide localized and controlled oxygen delivery to hypoxic wound environments. (Desmet et al., 2018) However, these approaches remain predominantly at the preclinical stage.

Sodium percarbonate (SPC) has been investigated as an oxygen-generating material because it releases hydrogen peroxide in an aqueous environment, which subsequently decomposes to generate oxygen (Chandra et al., 2015). Zehra et al. incorporated SPC into polycaprolactone (PCL) electrospun nanofibers and demonstrated gradual oxygen release and improved vascularization and wound repair in experimental diabetic wounds (Zehra et al., 2020). These findings provide preclinical evidence for SPC-containing oxygen-releasing dressings, but their safety, optimal oxygen-delivery kinetics, manufacturing reproducibility, and clinical efficacy in patients with DFUs remain to be established. Thus, oxygen-generating nanomaterials should currently be regarded as investigational approaches rather than established clinical treatments.

#### Balancing the oxygen equation

Reactive oxygen species (ROS) have context-dependent effects during wound healing. Physiological levels of ROS participate in cell signaling and host defense, whereas excessive oxidative stress can contribute to persistent inflammation and impaired tissue repair in diabetic wounds. Nitric oxide (NO) is an important signaling molecule involved in angiogenesis, inflammation, and tissue repair, and impaired NO bioavailability has been reported in diabetes (Riccio and Schoenfisch, 2012). Consequently, several topical NO-delivery strategies, including organic nitrates, nitrites, metal–NO complexes, diazeniumdiolates (NONOates), and S-nitrosothiols (RSNOs), have been investigated.

Zhang et al. developed a copper-based metal–organic framework (HKUST-1) as an NO-loading vehicle and incorporated NO-loaded HKUST-1 into a coaxial nanofiber scaffold. The system enabled sustained NO release and, together with the release of copper ions, enhanced endothelial-cell growth, angiogenesis, collagen deposition, and anti-inflammatory responses in experimental diabetic wounds (Zhang et al., 2020). These findings represent preclinical evidence, based on cellular and animal experiments, rather than demonstrated human clinical efficacy. Other NO-releasing nanomaterials and microneedle-based systems have similarly shown wound-healing effects in diabetic animal models, but their clinical translation remains to be established (Zhang et al., 2020).

Overall, oxygen-generating and NO-delivery nanomaterials provide experimental strategies for modifying hypoxia, oxidative stress, angiogenesis, and inflammation in diabetic wounds. Nevertheless, most available evidence remains *in vitro* and/or animal-based, with limited or no adequately powered human clinical evidence for these specific nanotechnology platforms in DFUs. Important translational considerations include control of oxygen or NO release, dose-dependent toxicity, material stability, reproducibility, scalable manufacturing, sterilization, long-term biocompatibility, and regulatory requirements. Accordingly, preclinical findings should not be extrapolated directly to clinical effectiveness, and well-designed human studies are required to establish the safety and therapeutic benefit of these approaches in patients with DFUs.

#### Nano-drug delivery system

Nanoparticle-based drug-delivery systems are emerging approaches that may improve drug stability and facilitate controlled and prolonged release. These systems employ various biomaterials and have been investigated for their potential applications in diabetic wound healing. The reported applications and key findings of different nanoparticle systems are summarized in Table 6. There are various types of NDDSs, and they include liposomes, polymeric nanoparticles, inorganic nanoparticles, lipid nanoparticles, nanofibrous structures, and nanohydrogels (Figure 2).

**Table 6:** Nanoparticle-based systems investigated for diabetic wound healing.

Nanoparticle type	Application	Key findings	Evidence level	Ref.
Liposomes	Deformable liposomes	Improved drug-delivery efficiency and penetration across the viable epidermis	In vitro/ex vivo	Zhang and Michniak-Kohn (2020)
Polymeric nanoparticles	Controlled drug release	Melatonin-loaded lecithin–chitosan nanoparticles improved wound healing and tissue repair in diabetic rats	Animal study	Lopes Rocha Correa et al. (2020)
Inorganic nanoparticles	Quercetin-AgNPs	Demonstrated antimicrobial and antioxidant effects and improved wound healing in an experimental diabetic wound model	Animal study	Badhwar et al. (2021)
Inorganic nanoparticles	Multifunctional nanocarriers	A silver nanoparticle–based multifunctional formulation promoted wound closure and demonstrated antibacterial activity in an experimental diabetic wound model	Animal study	Choudhary et al. (2021)
Lipid nanoparticles	Topical treatment	Retinoic acid-loaded solid lipid nanoparticles improved wound closure and collagen deposition in diabetic mice; other lipid nanocarrier formulations demonstrated anti-inflammatory, proangiogenic, or wound-repair effects in experimental models	Predominantly animal/in vitro studies	Arantes et al. (2020);Sun et al. (2020)
Nanofibers	Drug delivery via electrospinning	Curcumin-, sesamol-, insulin-, antibiotic-, and growth-factor-loaded nanofibrous scaffolds promoted wound repair, re-epithelialization, angiogenesis, or modulation of inflammatory pathways in experimental models	Predominantly animal/in vitro studies	Agarwal et al. (2021);Liu et al. (2020);Lee et al. (2020)
Nanohydrogel	Moist environment and controlled delivery	Exosome- and growth-factor-containing nanohydrogels promoted cell proliferation, migration, angiogenesis, and wound healing in experimental diabetic wound models	Animal study	Zhang et al. (2021);Xiong et al. (2022)

0AgNPs, silver nanoparticles; SLNs, solid lipid nanoparticles; LNPs, lipid nanoparticles; MMP9, matrix metalloproteinase-9; TGF-β, transforming growth factor- β; BMSCs, bone marrow stem cells; PLGA, poly (lactic-co-glycolic acid); GS, gentamicin sulfate; rhEGF, recombinant human epidermal growth factor; PVA, polyvinyl alcohol gel; ERK, extracellular signal-regulated kinase.

**Figure 2:**
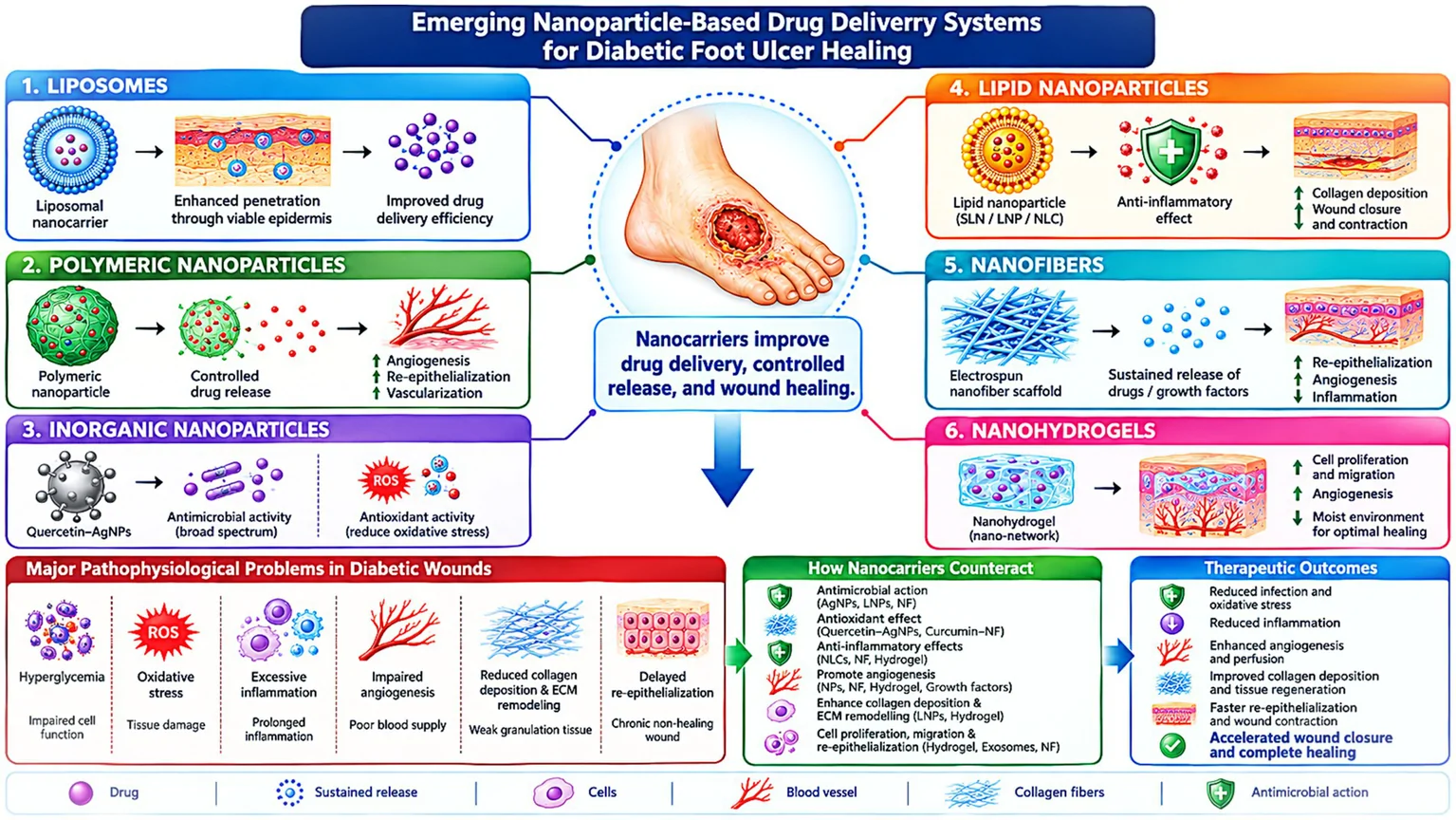
Emerging therapeutic nanoparticles in treating diabetic foot ulcer.

Overall, the studies summarized in this table are predominantly preclinical, comprising *in vitro*/ex vivo and animal studies; no human clinical evidence is presented for these specific formulations. Therefore, their reported wound-healing, antimicrobial, or regenerative effects should not be interpreted as established clinical efficacy in DFUs.

## Conclusion

Effective management of DFUs requires a comprehensive, multidisciplinary approach addressing impaired wound healing, infection, pressure, vascular insufficiency, and metabolic control. Established components of care, including pressure off-loading, regular debridement, appropriate infection management, glycaemic optimization, and vascular assessment and restoration when indicated, remain the foundation of DFU treatment. Advanced wound dressings and negative pressure wound therapy may provide additional benefits in selected patients.

Clinically available antimicrobial agents remain important for the management of DFIs, with antibiotic selection guided by infection severity, likely or documented pathogens, antimicrobial susceptibility, patient factors, and local resistance patterns. Newer antimicrobial agents may offer treatment options for selected infections caused by resistant organisms; however, their use should be individualized and align with antimicrobial stewardship principles. Biofilm-associated tolerance should also be distinguished from genetically mediated antimicrobial resistance when considering treatment response.

Nanotechnology and regenerative approaches, including growth-factor delivery systems, gene and RNA-based therapies, stem cell- and exosome-based interventions, oxygen-delivery biomaterials, and nano-enabled drug-delivery systems, represent emerging strategies for DFU management. These approaches have shown promise for drug delivery, inflammation, angiogenesis, tissue regeneration, and wound closure, but evidence for many remains predominantly *in vitro* or preclinical, with limited human clinical data. Their safety, reproducibility, scalability, manufacturing requirements, and regulatory pathways therefore require further evaluation before routine clinical implementation.

Overall, current DFU management should continue to prioritize established multidisciplinary care and evidence-based antimicrobial therapy, while emerging nanotechnology and regenerative interventions should be regarded as investigational or adjunctive approaches pending stronger clinical evidence. Well-designed clinical trials are needed to determine their safety, efficacy, long-term outcomes, and appropriate integration into standard DFU care. Such evidence will help define the potential role of these emerging technologies in improving wound healing and reducing infection-related complications and amputation in patients with DFUs.
